# Accuracy of endometrial length measurement in predicting IVF/ICSI outcome

**DOI:** 10.25122/jml-2021-0430

**Published:** 2022-09

**Authors:** Shaymaa Abdul-Sattar Nayyef, Thuraya Husamuldeen Abdullah, Manal Taha Al Obaidi

**Affiliations:** 1 Department of Obstetrics and Gynecology, Al-Yarmuk Teaching Hospital, Baghdad, Iraq; 2 Department of Obstetrics and Gynecology, Alimamain Alkathmain Medical City, Baghdad, Iraq; 3 High Institute for Infertility Diagnosis & Assisted Reproductive Technologies (ART), Baghdad, Iraq

**Keywords:** endometrial length, IVF/ICSI outcome, vaginal sonography

## Abstract

This study aimed to evaluate if endometrial length (EL) affects the likelihood of a positive pregnancy test in individuals who had *in vitro* fertilization/intra cytoplasmic sperm injection (IVF/ICSI). Between December 2020 and June 2021, 100 women from those referred to the High Institute for Infertility Diagnosis and Assisted Reproductive Technologies, Al-Nahrain University, for IVF/ICSI were selected. These women were 20–40 years old with primary type sub-fertility and met the inclusion criteria. Controlled ovarian hyperstimulation (antagonist protocol), followed by transvaginal sonography (TVS), was used to measure the endometrial length (EL) from the internal ostium to the fundus on the day of ovum collection. Afterwards, the association between endometrial length and the outcome was examined. The data were analyzed using the Statistical Package for Social Sciences (SPSS). The data show an important link between EL and treatment success. Endometrial length of ≥39 mm was considered a good cut-off point for IVF/ICSI pregnancy rates with a sensitivity of 75%, a specificity of 68%, a positive predictive value of 57%, and a negative predictive value of 83, and area under the curve of 0.81. An endometrial length of 39 mm can be used as an appropriate cut-off point in IVF/ICSI to predict a greater pregnancy rate. Vaginal sonography could be used in the ART cycle to measure the endometrial length and thickness and to determine endometrial receptiveness.

## INTRODUCTION

Conception delay, affecting 10 to 15% of reproductive-aged couples, is one of the most common reasons women seek medical attention to determine the cause and appropriate treatment [1]. IVF procedures, ovarian hyperstimulation regimens, and embryo transfer have all improved in recent years, but the pregnancy rates did not follow the upward trend. The quality of the embryo and the receptiveness of the endometrium are two of the most important variables in establishing pregnancy [2]. Good quality embryos and endometrium with high receptivity are both vital factors for human implantation to be successful [3]. When it comes to treating infertility, the implant is the weakest link. The endometrium is a receptor organ with dynamic tissue that grows and differentiates in response to most of the hormones implicated in the reproductive system [4]. Thus, the study of morphology and vascularity is believed to clarify the implantation failure riddle.

Consequently, as we follow the follicle growth, it is necessary to assess the endometrium by transvaginal sonography and color Doppler ultrasound as an earlier step to hCG triggering through any assisted reproductive technologies (ART) [5]. Various uterine characteristics linked to endometrial receptivity and their influence on ART outcomes have been evaluated in the previous years. When considering uterine parameters detected by ultrasonography, in addition to endometrial thickness and pattern, other parameters such as endometrial volume and uterine and endometrial blood flow are reflected as implantation indicators in IVF-ICSI cycles [6]. Although several studies have covered the prognostic significance of endometrial characteristics (thickness and echo pattern) in terms of ART success, little is known about uterine length in women with normal uterus. Consequently, in some studies, the catheter, hysterometer or vaginal sonography were used for endometrial length measurement and to compare pregnancy rates between groups with different endometrial lengths. The results show a discrepancy in findings. Therefore, the idea to assess the link between the length of the endometrium (from the internal ostium of the cervix to the uterine fundus) and the pregnancy rate in women who undergo IVF/ICSI emerged [7].

## MATERIAL AND METHODS

The cross-sectional comparative study was carried out on 100 women who attended the High Institute for Infertility Diagnosis and Assisted Reproductive Technologies, Al-Nahrain University, for IVF/ICSI between December 2020 and June 2021. All women were nulliparous, 20–40 years of age and met the inclusion criteria: first trial of IVF/ICSI cycle reaching the day of embryo transfer with grade 1 (G1) embryos, no uterine or endometrial anomalies, no history of abortion, curettage, hysteroscopy or surgical intervention to uterus or endometrium. A flexible antagonist protocol was used, and all patients were expected to be normal responders (according to sonography and hormonal investigation on the second day of the cycle). FSH treatment started on the 2^nd^–3^rd^ day of menstruation, and then it was adjusted according to patient response, who was monitored by serial transvaginal sonography (TVS) and estrogen E2 level from day five and every 2–3 days. Once the leading follicles got to the diameter of 14 mm, 0.25 mg of GnRH antagonist (cetrorelix, Cetrotide; Merck-Serono) was given daily until the day of trigger (by hCG, when the patient had at least three follicles that were ≥18 mm). Oocytes retrieval was carried out 34–36 hours later, followed by a two-dimensional TVS scan to measure endometrial thickness, length (from internal os to the fundus) and sub endometrial blood flow color Doppler indices [resistance index RI and systolic to diastolic ratio (S/D ratio)], by the 6 MHz vaginal probe of SonoAce-X6 Ultrasound Set (Medison, Seoul, South Korea). There was an assessment of weight and height to obtain body mass index (BMI), and the E2 level was measured on the day of the trigger. To confirm clinical pregnancy, a minimum of one gestational sac with positive fetal heart activity detected by ultrasound was mandatory.

### Statistical analysis

The data were analyzed using Statistical Package for Social Sciences (SPSS). Statistical parameters including frequency, mean and standard deviation were calculated to describe the sample. The groups were compared based on an independent sample t-test (unpaired t-test between two groups of continuous variables). The coefficient of Pearson's correlation (r) was considered for calculating the degree of association among continuous parameters. The receiver operating characteristics (ROC) curve was used to determine the cut-off value, sensitivity, and specificity, and the findings were declared statistically significant when the p-value was less than 0.05.

## RESULTS

### Baseline information and pregnancy rate of the participants

The pregnancy rate for the 100 women enrolled in this study was 36%. No important difference was found between pregnant and non-pregnant groups regarding the mean age of the patients (p=0.194), mean body mass index (BMI) (p=0.220), causes of infertility (p=0.133), duration of infertility (p=0.492), E2 on the day of trigger (p=0.332) and duration of treatment (p=0.546). Furthermore, we found no significant difference in total oocyte count (p=0.341) and the number of embryo transfers (p=0.281) (Table 1).

**Table 1:** Demographic and clinical parameters of pregnant and non-pregnant females.

Parameter (Mean±SD)	Pregnant females (36)	Non-Pregnant females (64)	P-value
Age (years)	29.67±4.99	31.13±5.56	0.194
BMI (kg/m^2^)	28.11±5.36	29.28±4.04	0.220
Duration of infertility (years)	8.81±4.18	8.15±4.73	0.492
E2 on the day of trigger (pg/ml)	1532±784	1394±607	0.332
Duration of treatment (days)	8.72±1.41	8.30±1.42	0.546
Total oocytes count	12.08±5.38	10.94±5.95	0.341
Total transferred embryos	3.14±0.83	2.94±0.92	0.281

* – p-value<0.05 (significant); SD – Standard deviation; BMI – Body mass index.

### Comparison of endometrial length between two groups

Regarding the EL, there is a significant difference between the two groups (<0.001), with no significant difference in endometrial thickness, resistance index (RI), and systolic to diastolic (S/D) ratio (Table 2).

**Table 2:** Comparison of sonographic measures and blood flow indices between pregnant and non-pregnant females.

Parameter	Pregnant females	Non-Pregnant females	P-value
Endometrial length (mm)	41.64±4.03	36.22±4.57	<0.001*
Endometrial thickness (mm)	9.53±1.54	9.80±1.68	0.414
Resistance index	0.44±0.11	0.48±0.09	0.085
Systolic to diastolic ratio	1.98±0.52	2.09±0.49	0.263

* – p-value<0.05 (significant).

### Correlation between endometrial length and other parameters

**Figure 1:**
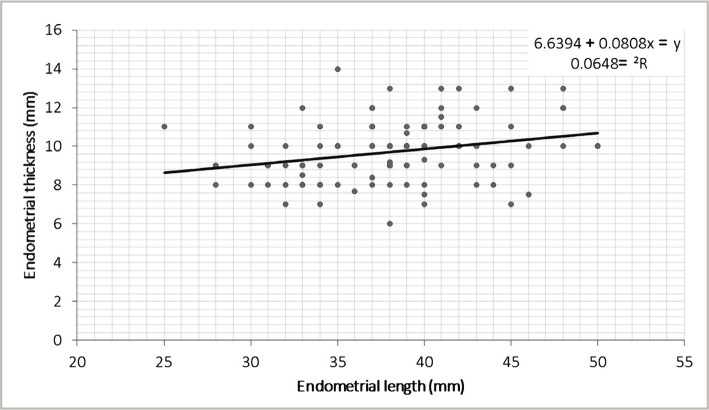
Correlation between endometrial length and thickness.

There was a positive significant correlation between endometrial length and endometrial thickness (r=0.270, p=0.007) (Figure 1), and adverse association with systolic to diastolic ratio (r=-0.226, p=0.024). Moreover, there was a significant positive correlation concerning endometrial resistance index with mean patient's age (r=0.221, p=0.028) and systolic to diastolic (S/D) ratio (r=0.894, p<0.001) (Table 3).

**Table 3:** Correlation between endometrial length, thickness, and blood flow indices with patient's parameters.

Parameters		Endometrial length	Endometrial thickness	Resistance index (RI)	S/D ratio
**Endometrial length**	R	1	0.270	-0.191	-0.226
	p-value	-	0.007*	0.059	0.024*
**Endometrial thickness**	R	0.270	1	-0.002	-0.025
	p-value	0.007*	-	0.986	0.807
**Resistance index**	R	-0.191	-0.002	1	0.894
	p-value	0.059	0.986	-	<0.001*
**Systolic to diastolic (S/D) ratio**	R	-0.226	-0.025	0.894	1
	p-value	0.024*	0.807	<0.001*	-
**E2 on the day of trigger**	R	0.048	0.040	-0.025	-0.081
	p-value	0.633	0.690	0.809	0.423
**Age**	R	-0.043	0.049	0.221	0.138
	p-value	0.669	0.630	0.028 *	0.172
**BMI**	R	-0.124	0.022	-0.064	-0.112
	p-value	0.218	0.825	0.531	0.267
**Weight**	R	-0.069	0.018	-0.113	-0.164
	p-value	0.496	0.856	0.265	0.103
**Height**	R	0.087	0.003	-0.180	-0.185
	p-value	0.390	0.978	0.075	0.065
**Duration of treatment**	R	0.016	0.076	-0.001	-0.012
	p-value	0.877	0.450	0.990	0.906

R – Pearson's correlation coefficient; * – p-value<0.05 (significant).

### Cut-off value of endometrial length

**Figure 2:**
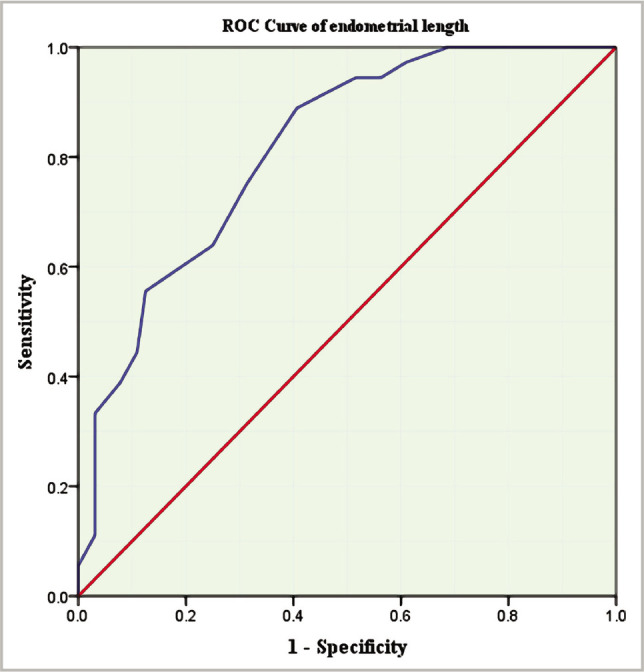
ROC curve of endometrial length as a predictor of positive pregnancy.

The receiver operating characteristic curve was used to calculate the cut-off value of endometrial length as a predictor of positive pregnancy with satisfactory sensitivity, specificity, and area under the curve. According to the results, the cut-off value of endometrial length was ≥39 mm with sensitivity=75%, specificity=68.8% and area under curve=0.814. The positive predictive value was 57.4%, while the negative predictive value was 83.0% (Figure 2).

## DISCUSSION

Technological advances in ultrasound imaging have improved the understanding of the endometrium cyclic alterations and raised the possibility of measuring the likelihood of embryo implantation in both natural and induced cycles. To better comprehend and describe the endometrial circumstances necessary for embryo implantation and pregnancy, new imaging methods are being developed [8]. Because infertility treatment is expensive and time-consuming, and implantation rates are low, it is critical to find a technique to predict the success of ART treatments before they begin [9]. For that, the goal of this study was to see if there was a link between endometrial length and pregnancy outcome in the IVF-ICSI protocol. There are differences in measures of endometrial length between studies. In the present study, we measured endometrial length from internal ostium to fundus (excluding cervix length), so the mean of EL was 41.6 mm in pregnant women and 36.2 mm in non-pregnant women.

In contrast, some other studies measure it from external os to fundus, so the mean of EL was 70–90 mm. We consider our assessment and its relationship with ART outcome as more reasonable. A positive correlation was found between endometrial length and positive pregnancy rates. This is similar to Sang Sik Chun et al., who found that the correlation between length of the uterine cavity (measured from the fundus to external os) and pregnancy rates was positive, *i.e*., longer uterine cavity lengths were associated with greater clinical pregnancy rates in IVF-ICSI patients. There is a good possibility that an increased uterine cavity length correlates with an endometrial volume increase, which contributes to higher pregnancy rates [2].

In agreement with our study, Firoozeh Ahmadi et al. assessed the relationship between endometrial length (measured by ultrasound from the internal os to the fundus) and cycle outcome and revealed an important relationship between the length of endometrium and success of treatment, suggesting that 41 mm (EL) with acceptable sensitivity and specificity can be applied as a suitable cut-off point for IVF trial prediction [7]. The likelihood of having a live birth rises by 3.59 for each additional centimeter of endometrial cavity length (ECL) after treatment with estrogen in recipients with ideal thickness of endometrial cavity (ECT) using donor oocytes, as revealed in another study [10]. This explains why a prolonged estradiol treatment for embryo transfer preparation may be required to produce accurate ECL rather than merely an ECT. The relation between IVF treatment and uterine depth (endometrial length) was also evaluated by Williams et al., who stated that the depth of the uterine cavity was meaningfully increased by stimulation for IVF. Identifying the correct depth of placement during embryo transfer should not depend on pre-stimulation uterine sounding dimensions [11].

In contrast, Egbase et al. believed that uterine cavity length was not significantly affected by long-term GnRH agonist administration. They also looked at how the length of the uterine cavity affected IVF implantation and pregnancy rates and found that women who have uterine cavity lengths (from external os to fundus) of 7–9 cm had higher pregnancy rates than those with uterine cavity lengths of <7 or >9 cm, but not statistically significant differences were found [12]. In a study by L.K. Hawkins and coworkers, the authors found that women with extreme uterine lengths (<7 or >9 cm) have decreased probability of having a live delivery, while those with a uterine length less than 6 cm have a high probability for spontaneous abortions [13]. The depth at which embryos are placed in the uterus is an important aspect of embryo transfer (around 1 cm distal to the fundus), according to Foran and colleagues. Endometrial cavity lengths (ECLs) were measured at three different times: (1) before the stimulation cycle using catheterized trial embryo transfer (TET), (2) at the commencement of the stimulation or baseline (BL), focusing on the suppression by Gonadotropin-releasing hormone agonist to assess the endometrial cavity length by sonography, and (3) a stimulated per ovulatory (PO) ultrasound exams a third way to find out. According to the results, means of ECL were TET 7.3 cm, baseline 6.7 cm, and per ovulatory 7.8 cm; all had substantially diverse mean ECL values of p=0.0001. The average difference between PO and BL measurements was 1.1 cm (p=0.0001); with the cycle proceeding, the endometrial cavity increases in length and size. It is possible that the TET measurement is incorrect when ECLs are utilized to assess embryo location around 1 cm distal to the fundus at ET. Because doctors can see where to best put the embryos, ultrasonography can increase the chances of getting pregnant [14].

According to S. Bassil et al., in a study between conception and non-conception cycles, there were no differences in endometrial thickness, width, length, or pattern. There was no effect on the pregnancy outcome from endometrial development and pattern transformation during stimulation [15]. However, the present study shows an insignificant positive association between endometrial length (EL) and a woman's height, and insignificant inverse relation with age, weight, and BMI. One study showed that the uterine length increases with age in the 21–40 age group and decreases in the 41–60 age group. Parous but not nulliparous women show a positive connection between uterus length and age, body weight, height, and surface area. With the same age, weight, height, and body surface area, the uterine length was shorter in nulliparous women than in parous women [16]. Existing data show no significant difference between the pregnant and non-pregnant groups in terms of endometrial thickness. This is in line with the Julian A. et al. research, which found that endometrial thickness was not substantially related to clinical outcomes of euploid embryo transfer, but the endometrial pattern was [17]. Furthermore, the current study demonstrates a positive relationship between age and the resistant index (RI), while E2, BMI, weight, height, and the duration of treatment with RI and the S/D ratio have a negligible inverse relationship. Ernest Hung Yu Ng et al. showed that age, smoking habits, infertility types, parity, and reasons for subfertility did not influence the endometrial and sub endometrial 3D power Doppler flow findings during IVF therapy. Nonetheless, they found that the serum E2 concentration and the endometrial flow index FI (r=-0.109; p=0.006) were negatively related [18].

## CONCLUSIONS

The present study revealed a substantial positive relationship between endometrial length and thickness. This can be explained by the effect of hormonal treatment on the endometrium, as it increases both endometrial thickness and length. This effect may be related to the dose and duration of therapy. Moreover, there is a significant inverse correlation between endometrial length and S/D ratio. In our opinion, an increase in endometrial length may be associated with an increase in vascularity, and so does the decrease in the S/D ratio.

## Personal thanks

Our thanks to all staff members of the High Institute for Infertility, Diagnosis and Assisted Reproductive Technologies for their support and facilitation of work for choosing patients, performing the procedure and making it easy and possible to manage and follow up with patients (Dr.Raid, Ramih, Mariam).
